# Comprehensive analysis of the association between triglyceride-glucose index and coronary artery disease severity across different glucose metabolism states: a large-scale cross-sectional study from an Asian cohort

**DOI:** 10.1186/s12933-024-02355-3

**Published:** 2024-07-13

**Authors:** Sheng Zhao, Zuoxiang Wang, Ping Qing, Minghui Li, Qingrong Liu, Xuejie Pang, Keke Wang, Xiaojin Gao, Jie Zhao, Yongjian Wu

**Affiliations:** 1 Department of Cardiology, State Key Laboratory of Cardiovascular Disease, National Center for Cardiovascular Diseases, Fuwai Hospital, Chinese Academy of Medical Sciences and Peking Union Medical College, Beijing, China; 2 Department of Cardiology, Beijing Aerospace General Hospital, Beijing, China; 3 Department of Respiratory and Critical Care, Chinese PLA General Hospital, the First Medical Centre, Beijing, China; 4 Department of Cardiology, Chinese PLA General Hospital, the Second Medical Centre, Beijing, China; 5 State Key Laboratory of Cardiovascular Disease, Fuwai Hospital, National Center for Cardiovascular Disease, Chinese Academy of Medical Sciences and Peking Union Medical College, No.167 North Lishi Road, Xicheng District, 100037 Beijing, China

**Keywords:** Triglyceride-glucose index, Coronary artery disease, Glucose metabolism state, Diabetes mellitus

## Abstract

**Background:**

The triglyceride-glucose (TyG) index is associated with the development and prognosis of coronary artery disease (CAD). However, the impact of the TyG index on CAD severity across different glucose metabolism states exhibits significant disparities in previous research.

**Methods:**

This cross-sectional study comprised 10,433 participants from a prospective cohort. Participants were categorized into four groups based on glucose metabolism state: normal glucose regulation (NGR), prediabetes (pre-DM), diabetes mellitus (DM) without insulin prescribed (Rx), and DM with insulin Rx. The TyG index was determined by the following formula: Ln [TG (mg/dL) × FPG (mg/dL) / 2], where TG is triglycerides and FPG is fasting plasm glucose. Statistical methods such as binary logistic regression, interaction analysis, restricted cubic spline (RCS), and receiver operating characteristic (ROC) were employed to analyze the relationship between the TyG index and CAD severity across the entire population and glucose metabolism subgroups. Mediation analysis was conducted to examine the mediating effects of glycated hemoglobin (HbA1c) on these relationships. Sensitivity analysis was performed to ensure the robustness of the findings.

**Results:**

Multivariable logistic regression analysis revealed a significant positive association between the TyG index and multi-vessel CAD in the entire population (OR: 1.34; 95% CI: 1.22–1.47 per 1-unit increment). Subgroup analysis demonstrated consistent positive associations in the NGR, pre-DM, and DM non-insulin Rx groups, with the highest OR observed in the NGR group (OR: 1.67; 95% CI: 1.3–2.14 per 1-unit increment). No correlation was found in the DM with insulin Rx subgroup. RCS analyses indicated the distinct dose-response relationships across different glucose metabolism subgroups. Including the TyG index in the established model slightly improved the predictive accuracy, particularly in the NGR group. Mediation analyses showed varying mediating effects of HbA1c among different glucose metabolism subgroups. Sensitivity analysis confirmed the robustness of the aforementioned relationships in the new-onset CAD population and in individuals not using antilipidemic medications.

**Conclusions:**

The TyG index positively associated with CAD severity across all glucose metabolism states, except for individuals receiving insulin treatment. Moreover, it might serve as a supplementary noninvasive predictor of CAD severity in addition to established factors, especially in NGR patients.

**Graphical abstract:**

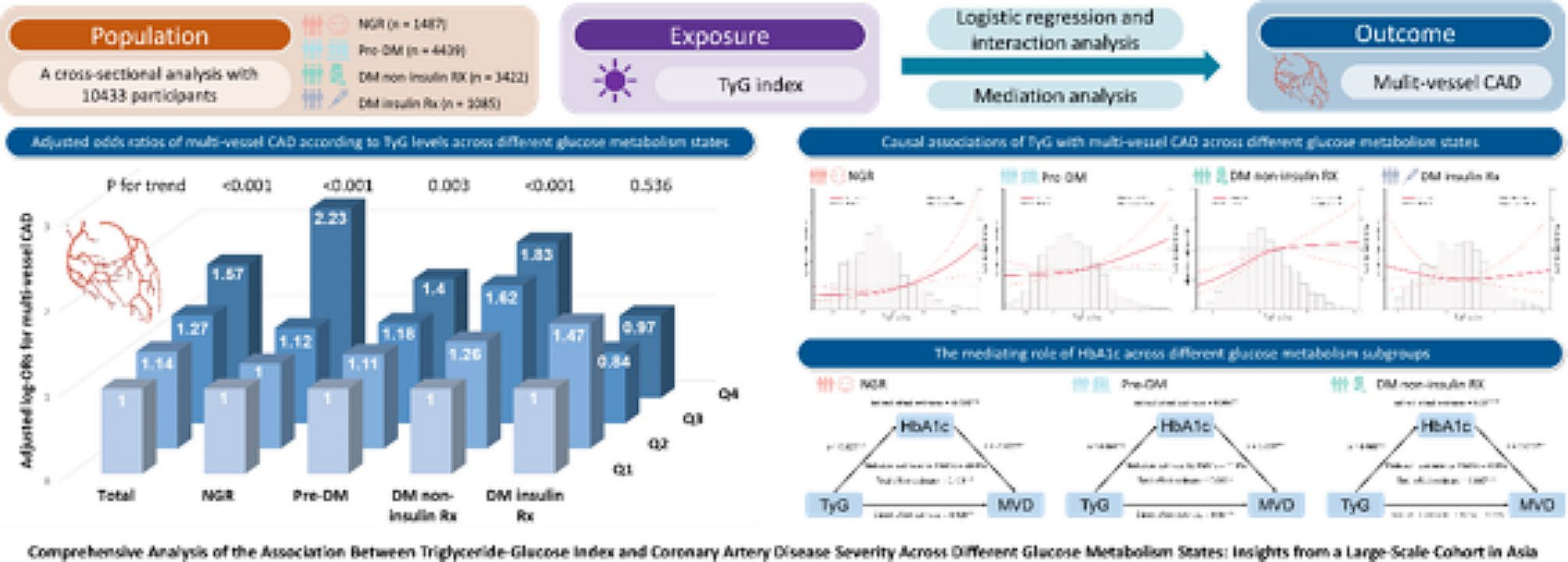

**Supplementary Information:**

The online version contains supplementary material available at 10.1186/s12933-024-02355-3.

## Introduction

Despite advancements in healthcare, coronary artery disease (CAD) remains the primary cause of global mortality due to factors such as aging, rising rates of diabetes, obesity, and unhealthy lifestyles [1]. The severity of CAD, primarily assessed based on the number of stenotic vessels diagnosed by coronary artery angiography (CAG), profoundly impacts clinical management and prognosis. Compared to individuals with single-vessel CAD, those with multi-vessel CAD are at increased risk of heart failure, comorbidities, and major adverse cardiovascular events (MACEs), especially in the presence of abnormal glucose metabolism [2–6]. Moreover, multi-vessel CAD warrants more intricate considerations in devising revascularization strategies and pharmacological interventions [7–9]. Given the high prevalence, unfavorable prognosis, and intricate therapeutic challenges associated with multi-vessel CAD, there is a pressing need for biomarkers to facilitate early detection of affected individuals.

Insulin resistance (IR) is a primary feature of abnormal glucose metabolism and has been established as a pivotal contributor to CAD across various pathological processes [10, 11]. The triglyceride–glucose (TyG) index, recognized as a dependable surrogate indicator of IR, correlates with both the progression and prognosis of CAD [12–14]. The TyG index is easily accessible and cost-effective, making it a promising tool for clinical management of CAD [15]. Recent studies have demonstrated an association between the TyG index and multi-vessel CAD. However, findings from these studies have shown inconsistencies across different glucose metabolism subgroups and, in some cases, conflicting results [16–18]. A most recent small-scale study has even negated this association in new-onset CAD population, irrespective of their glucose metabolism states [15].

Therefore, to address the controversy, we refined population categorization based on glucose metabolism and utilized a larger sample size to comprehensively investigate the association between the TyG index and CAD severity.

## Methods

### Study design and population

**Fig. 1:**
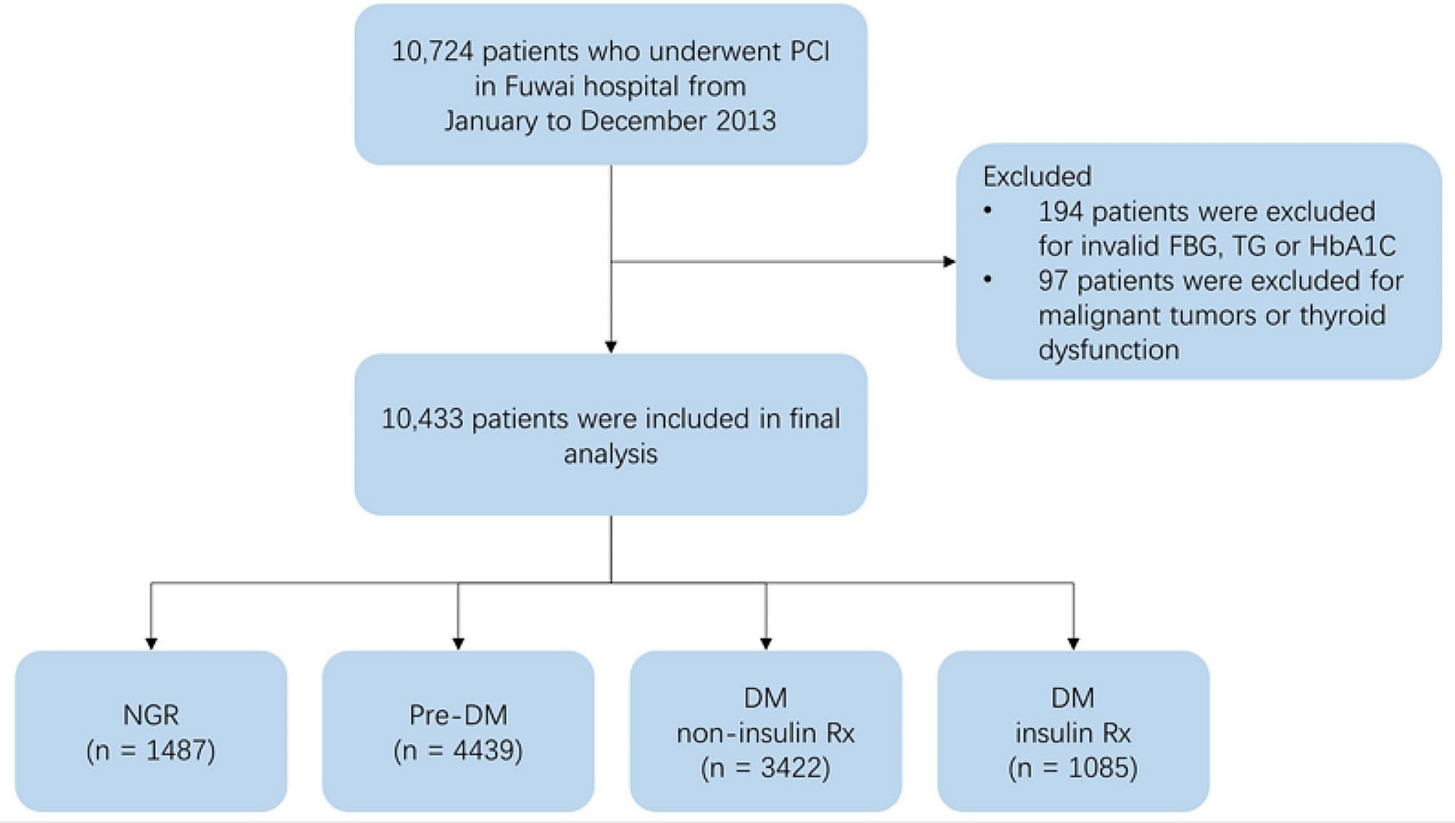
Flowchart. FBG: fasting blood glucose; HbA1c: glycated hemoglobin A1c; PCI: percutaneous coronary intervention; DM: diabetes mellitus; NGR: normal glucose regulation; pre-DM: prediabetes; Rx: prescription; TG: triglycerides

This cross-sectional study encompassed 10,433 participants from a prospective cohort conducted at Fuwai Hospital, National Center for Cardiovascular Diseases in Beijing, China. The research protocol adhered strictly to the principles outlined in the Declaration of Helsinki and received formal approval from the Fuwai Hospital Ethics Review Committee. Prior to enrollment, all participants provided informed consent. Between January 1, 2013, and December 31, 2013, a total of 10,724 patients who underwent percutaneous coronary intervention (PCI) at Fuwai Hospital were subjected to consecutive screening. The indications for PCI included symptomatic coronary artery disease, evidence of myocardial ischemia, and significant stenosis observed on coronary angiography, based on contemporary clinical guidelines and local standard practice. Exclusion criteria included: (1) missing important data such as fasting plasm glucose (FPG), triglycerides (TG), hemoglobin A1c (HbA1c), and (2) comorbidities such as malignant tumors, uncontrolled thyroid dysfunction. Ultimately, 10,433 patients were enrolled and categorized into four subgroups based on glucose metabolism state: normal glucose regulation (NGR), prediabetes (pre-DM), diabetes mellitus (DM) without insulin prescribed (Rx), and DM with insulin Rx. The detailed population enrollment process is illustrated in Fig. 1.

### Data collection and definitions

We collected baseline demographic and clinical data prospectively for all patients. Demographic information included age, sex, body mass index (BMI) and smoking status. Clinical data included laboratory test results, auxiliary examination findings, CAG findings and current medication. FPG and other biochemical indicators were assessed using the LABOSPECT 008 system (Hitachi, Tokyo, Japan), and HbA1c was determined using high-performance liquid chromatography (G8; TOSOH, Tokyo, Japan). BMI was determined by dividing weight (in kilograms) by the square of height (in meters). The estimated glomerular filtration rate (eGFR) was assessed using the Chronic Kidney Disease Epidemiology Collaboration creatinine equation [19]. The TyG index was calculated using the following formula: Ln [TG (mg/dL)×FPG (mg/dL) / 2]. CAD was defined as the presence of at least one major coronary artery with ≥ 50% stenosis as determined by CAG, which included the left anterior descending, left circumflex, and right coronary artery. The severity of CAD was gauged by the number of coronary arteries exhibiting ≥ 50% stenosis. Single-vessel CAD was diagnosed if only one major artery showed ≥ 50% stenosis, while multi-vessel CAD required ≥ 50% stenosis in two or more arteries. Notably, ≥ 50% stenosis in the left main coronary artery indicated multi-vessel CAD due to its anatomical significance. The aforementioned assessment was conducted by at least two intervention specialists following the completion of CAG. Hypertension is defined as a systolic blood pressure (SBP) ≥ 140 mmHg and/or diastolic blood pressure (DBP) ≥ 90 mmHg observed on more than two occasions during the baseline hospitalization or in individuals receiving antihypertensive medications, or based on previous diagnosis. Dyslipidemia is defined as having plasma total cholesterol (TC) ≥ 5.2 mmol/L, TG ≥ 1.7 mmol/L, low-density lipoprotein cholesterol (LDL-C) ≥ 3.4 mmol/L, high-density lipoprotein cholesterol (HDL-C) < 1.0 mmol/L, and/or individuals receiving lipid-lowering therapy, or based on previous diagnosis. The glucose metabolism state was diagnosed according to the latest American Diabetes Association (ADA) “Standards of Care in Diabetes”, taking into account FPG and HbA1c levels measured during hospitalization, while also considering self-reported prior diagnosis and medication [20]. It is noteworthy that, given the potential impact of insulin on IR and the inconsistent conclusions of previous studies on different glucose metabolism subgroups (NGR, pre-DM and DM), further differentiation of the DM population was made based on whether insulin treatment was prescribed (DM non-insulin Rx and DM insulin Rx).

### Statistical analysis

Continuous variables are expressed as the mean ± standard deviation (SD) or as the median and interquartile range (IQR), while categorical variables are presented as numbers and percentages. Statistical analyses included the χ2 test for comparing categorical variables and the t test, analysis of variance, Mann–Whitney U test, or Kruskal–Wallis H test for continuous variables. Participants’ baseline characteristics were delineated based on quartiles of the TyG index, CAD severity, and glucose metabolism states, respectively. Kernel density estimations were used to describe the distribution of TyG index by glucose metabolism states. The relationship between the TyG index and multi-vessel CAD in the entire population and among different glucose metabolism states was investigated with logistic regression analysis in the crude model and adjusted model. The adjusted model was adjusted for age, sex, BMI, SBP, current smoking status, hypertension, dyslipidemia, eGFR, left ventricular ejection fraction (LVEF), HDL-C, LDL-C, lipoprotein(a) [Lp(a)], and current medication with antidiabetic (only for the overall population), antihypertensive, antiplatelet, and antilipidemic drugs. Correlation results were expressed as odds ratios (OR) and 95% confidence intervals (CI). The interaction of the TyG index with different glucose metabolism states on multi-vessel CAD was assessed by including stratification analysis and interaction tests in the regression model. To account for the dose–response relationship (linear or nonlinear) between TyG index and multi-vessel CAD, restricted cubic spline (RCS) curves adjusted for the same variables as in adjusted model were performed with 3 knots. Non-linearity tests were performed using the likelihood ratio test. Receiver operating characteristic (ROC) curves were used for diagnostic value analysis, and the incremental predictive performance for multi-vessel CAD after introducing the TyG index to the established risk model with fully adjusted variables was evaluated by calculating the C-statistic, continuous net reclassification improvement (NRI), and integrated discrimination improvement (IDI). In addition, mediation analyses were employed to investigate whether the association between TyG index and multi-vessel CAD could be explained by HbA1c after adjusting for factors in the adjusted model. Sensitivity analysis was performed in the new-onset CAD population. All the statistical analyses were performed using R version 4.3.0 software (R Foundation for Statistical Computing, Vienna, Austria), with statistical significance set at a P value < 0.05.

## Results

### Baseline characteristics

A total of 10,433 participants with CAD were included. Mean age was 58.3 ± 10.3 years, with 8053 (77.2%) males. Mean BMI was 25.9 ± 3.2 kg/m^2^. Among them, 1487 (14.3%) presented with NGR, 4439 (42.5%) presented with pre-DM, 3422 (32.8%) presented with DM non-insulin Rx, and 1085 (10.4%) presented with DM insulin Rx. The median (IQR) TyG index was 8.89 (8.54, 9.28) in the overall population, with 75.8% of participants exhibiting multi-vessel CAD. Patients were stratified into four quartiles based on the TyG index: the Q1 group (TYG ≤ 8.54), *n* = 2608; the Q2 group (8.54 < TYG ≤ 8.89), *n* = 2608; the Q3 group (8.89 < TYG ≤ 9.28), *n* = 2608; the Q4 group (TYG > 9.28), *n* = 2609. Participants with higher TyG index were more likely to be younger, female, higher levels of BMI, higher blood pressure, hypertension, dyslipidemia, DM, TG, TC, LDL-C, FPG, HbA1c, antidiabetic drugs, antihypertensive drugs and antilipidemic drugs, and lower levels of LVEF, HDL-C, Lp(a). According to the severity of CAD, participants were categorized into single-vessel and multi-vessel CAD groups. Those with multi-vessel CAD were older and had higher levels of BMI, SBP, current smoking, hypertension, dyslipidemia, DM, TG, TC, LDL-C, Lp(a), FPG, HbA1c, and greater usage of antidiabetic, antihypertensive, antiplatelet, and antilipidemic drugs. They also had lower eGFR, LVEF, and a lower incidence of new-onset CAD. The detailed baseline characteristics are presented in Table 1 for TyG index quartiles, Table 2 for CAD severity, and Table 3 for glucose metabolism states.

**Table 1:** Baseline characteristics according to TyG index quartiles

Variables	Total (*n* = 10,433)	TyG index quartiles				
		Q1 (*n* = 2608)	Q2 (*n* = 2608)	Q3 (*n* = 2608)	Q4 (*n* = 2609)	*P* value
TyG index	8.89 (8.54, 9.28)	8.30 (8.13, 8.43)	8.72 (8.63, 8.80)	9.06 (8.97, 9.16)	9.60 (9.42, 9.90)	< 0.001
Age, year	58.3 ± 10.3	60.1 ± 10.3	58.5 ± 10.3	57.7 ± 10.1	56.9 ± 10.0	< 0.001
Male, n (%)	8053 (77.2)	2077 (79.6)	2028 (77.8)	1999 (76.6)	1949 (74.7)	< 0.001
BMI, kg/m^2^	25.9 ± 3.2	25.0 ± 3.3	25.8 ± 3.0	26.4 ± 3.1	26.6 ± 3.1	< 0.001
SBP, mmHg	127.1 ± 16.5	126.4 ± 16.5	126.7 ± 16.2	127.2 ± 16.6	128.0 ± 16.6	0.004
DBP, mmHg	77.5 ± 10.4	76.6 ± 10.1	77.3 ± 10.1	77.9 ± 10.6	78.3 ± 10.6	< 0.001
Current smoker, n (%)	5963 (57.2)	1455 (55.8)	1458 (55.9)	1486 (57)	1564 (59.9)	0.007
Hypertension, n (%)	6725 (64.5)	1599 (61.3)	1644 (63)	1708 (65.5)	1774 (68)	< 0.001
Dyslipidemia, n (%)	7028 (67.4)	1567 (60.1)	1693 (64.9)	1815 (69.6)	1953 (74.9)	< 0.001
**Glucose metabolism state, n (%)**						< 0.001
NGR	1487 (14.3)	508 (19.5)	471 (18.1)	321 (12.3)	187 (7.2)	
Pre-DM	4439 (42.5)	1437 (55.1)	1259 (48.3)	1085 (41.6)	658 (25.2)	
DM non-insulin Rx	3422 (32.8)	505 (19.4)	706 (27.1)	953 (36.5)	1258 (48.2)	
DM insulin Rx	1085 (10.4)	158 (6.1)	172 (6.6)	249 (9.5)	506 (19.4)	
eGFR, ml/min	91.3 ± 15.0	91.4 ± 13.9	91.5 ± 14.5	91.4 ± 14.8	90.9 ± 16.5	0.464
LVEF, %	62.8 ± 7.3	63.4 ± 7.0	63.0 ± 7.1	62.7 ± 7.3	62.0 ± 7.5	< 0.001
TG, mmol/L	1.54 (1.15, 2.12)	0.97 (0.82, 1.12)	1.40 (1.24, 1.58)	1.85 (1.56, 2.12)	2.67 (2.09, 3.37)	< 0.001
TC, mmol/L	4.05 (3.44, 4.81)	3.59 (3.09, 4.21)	3.91 (3.39, 4.61)	4.21 (3.61, 4.92)	4.54 (3.89, 5.38)	< 0.001
HDL-C, mmol/L	0.99 (0.84, 1.17)	1.07 (0.90, 1.30)	1.01 (0.86, 1.19)	0.97 (0.83, 1.13)	0.93 (0.79, 1.09)	< 0.001
LDL-C, mmol/L	2.35 (1.86, 3.01)	2.05 (1.65, 2.56)	2.30 (1.84, 2.93)	2.52 (2.01, 3.17)	2.61 (2.05, 3.27)	< 0.001
Lp (a), mg/L	183.79 (78.22, 410.74)	194.26 (87.08, 427.50)	201.50 (87.62, 442.16)	188.93 (82.57, 417.62)	146.33 (60.31, 355.84)	< 0.001
FPG, mmol/L	5.55 (4.96, 6.81)	5.01 (4.63, 5.46)	5.35 (4.91, 6.01)	5.75 (5.13, 6.88)	7.36 (5.79, 9.74)	< 0.001
HbA1c, %	6.2 (5.8, 7.0)	6.0 (5.7, 6.3)	6.1 (5.8, 6.6)	6.30 (5.9, 7.0)	7.0 (6.2, 8.4)	< 0.001
New-onset CAD, n (%)	6669 (63.9)	1667 (63.9)	1685 (64.6)	1669 (64)	1648 (63.2)	0.756
Multi-vessel CAD, n (%)	7909 (75.8)	1853 (71.1)	1928 (73.9)	2001 (76.7)	2127 (81.5)	< 0.001
**Antidiabetic drugs, n (%)**						< 0.001
None	7871 (75.4)	2213 (84.9)	2150 (82.4)	1981 (76)	1527 (58.5)	
OHA	1477 (14.2)	237 (9.1)	286 (11)	378 (14.5)	576 (22.1)	
Insulin	1085 (10.4)	158 (6.1)	172 (6.6)	249 (9.5)	506 (19.4)	
Antihypertensive drugs, n (%)	2358 (22.6)	543 (20.8)	573 (22)	584 (22.4)	658 (25.2)	0.001
Antiplatelet drugs, n (%)	4625 (44.3)	1161 (44.5)	1120 (42.9)	1150 (44.1)	1194 (45.8)	0.231
Antilipidemic drugs, n (%)	5218 (50.0)	1213 (46.5)	1227 (47)	1296 (49.7)	1482 (56.8)	< 0.001

BMI: body mass index; CAD: coronary artery disease; DBP: diastolic blood pressure; DM: diabetes mellitus; eGFR: estimated glomerular filtration rate; FPG: fasting plasma glucose; HbA1c: glycated hemoglobin; HDL-C: high-density lipoprotein cholesterol; IR: insulin resistance; LDL-C: low-density lipoprotein cholesterol; Lp(a): lipoprotein(a); LVEF: left ventricular ejection fraction; NGR: normal glucose regulation; OHA: oral hypoglycemic agents; pre-DM: prediabetes; Rx: prescription; SBP: systolic blood pressure; TC: total cholesterol; TG: triglycerides; TyG: triglyceride-glucose

**Table 2:** Baseline characteristics according to CAD severity

Variables	Total (*n* = 10,433)	CAD severity		
		Single-vessel CAD (*n* = 2524)	Multi-vessel CAD (*n* = 7909)	*P* value
TYG index	8.89 (8.54, 9.28)	8.79 (8.47, 9.16)	8.92 (8.56, 9.32)	< 0.001
Age, year	58.3 ± 10.3	56.4 ± 10.4	58.9 ± 10.2	< 0.001
Male, n (%)	8053 (77.2)	1931 (76.5)	6122 (77.4)	0.348
BMI, kg/m^2^	25.9 ± 3.2	25.8 ± 3.2	26.0 ± 3.2	0.016
SBP, mmHg	127.1 ± 16.5	125.0 ± 15.7	127.7 ± 16.7	< 0.001
DBP, mmHg	77.5 ± 10.4	77.2 ± 10.3	77.6 ± 10.4	0.075
Current smoker, n (%)	5963 (57.2)	1377 (54.6)	4586 (58)	0.002
Hypertension, n (%)	6725 (64.5)	1456 (57.7)	5269 (66.6)	< 0.001
Dyslipidemia, n (%)	7028 (67.4)	1658 (65.7)	5370 (67.9)	0.039
**Glucose metabolism state, n (%)**				< 0.001
NGR	1487 (14.3)	492 (19.5)	995 (12.6)	
Pre-DM	4439 (42.5)	1188 (47.1)	3251 (41.1)	
DM non-insulin Rx	3422 (32.8)	673 (26.7)	2749 (34.8)	
DM insulin Rx	1085 (10.4)	171 (6.8)	914 (11.6)	
eGFR, ml/min	91.3 ± 15.0	93.1 ± 14.3	90.7 ± 15.1	< 0.001
LVEF, %	62.8 ± 7.3	63.2 ± 7.2	62.6 ± 7.3	0.001
TG, mmol/L	1.54 (1.15, 2.12)	1.46 (1.10, 2.03)	1.57 (1.17, 2.15)	< 0.001
TC, mmol/L	4.05 (3.44, 4.81)	3.99 (3.40, 4.70)	4.08 (3.46, 4.85)	< 0.001
HDL-C, mmol/L	0.99 (0.84, 1.17)	1.00 (0.84, 1.19)	0.99 (0.84, 1.17)	0.359
LDL-C, mmol/L	2.35 (1.86, 3.01)	2.28 (1.81, 2.91)	2.37 (1.87, 3.03)	< 0.001
Lp (a), mg/L	183.79 (78.22, 410.74)	165.44 (72.03, 371.14)	189.53 (80.85, 419.04)	< 0.001
FPG, mmol/L	5.55 (4.96, 6.81)	5.39 (4.87, 6.35)	5.62 (5.00, 6.98)	< 0.001
HbA1c, %	6.2 (5.8, 7.0)	6.10 (5.8, 6.6)	6.3 (5.9, 7.1)	< 0.001
New-onset CAD, n (%)	6669 (63.9)	1846 (73.1)	4823 (61)	< 0.001
**Antidiabetic drugs, n (%)**				< 0.001
None	7871 (75.4)	2109 (83.6)	5762 (72.9)	
OHA	1477 (14.2)	244 (9.7)	1233 (15.6)	
Insulin	1085 (10.4)	171 (6.8)	914 (11.6)	
Antihypertensive drugs, n (%)	2358 (22.6)	384 (15.2)	1974 (25)	< 0.001
Antiplatelet drugs, n (%)	4625 (44.3)	931 (36.9)	3694 (46.7)	< 0.001
Antilipidemic drugs, n (%)	5218 (50.0)	1005 (39.8)	4213 (53.3)	< 0.001

BMI: body mass index; CAD: coronary artery disease; DBP: diastolic blood pressure; DM: diabetes mellitus; eGFR: estimated glomerular filtration rate; FPG: fasting plasma glucose; HbA1c: glycated hemoglobin; HDL-C: high-density lipoprotein cholesterol; IR: insulin resistance; LDL-C: low-density lipoprotein cholesterol; Lp(a): lipoprotein(a); LVEF: left ventricular ejection fraction; NGR: normal glucose regulation; OHA: oral hypoglycemic agents; pre-DM: prediabetes; Rx: prescription; SBP: systolic blood pressure; TC: total cholesterol; TG: triglycerides; TyG: triglyceride-glucose

**Table 3:** Baseline characteristics according to glucose metabolism states

Variables	Total (*n* = 10,433)	Glucose metabolism states				
		NGR (*n* = 1487)	Pre-DM (*n* = 4439)	DM non-insulin Rx (*n* = 3422)	DM insulin Rx (*n* = 1085)	*P* value
TyG index	8.89 (8.54, 9.28)	8.71 (8.40, 9.00)	8.75 (8.44, 9.09)	9.08 (8.73, 9.50)	9.23 (8.78, 9.66)	< 0.001
Age, year	58.3 ± 10.3	54.5 ± 10.5	58.4 ± 10.3	59.6 ± 10.0	59.1 ± 9.5	< 0.001
Male, n (%)	8053 (77.2)	1256 (84.5)	3442 (77.5)	2548 (74.5)	807 (74.4)	< 0.001
BMI, kg/m^2^	25.9 ± 3.2	25.7 ± 3.3	25.7 ± 3.1	26.3 ± 3.2	26.3 ± 3.0	< 0.001
SBP, mmHg	127.1 ± 16.5	125.2 ± 15.9	126.4 ± 16.5	128.1 ± 16.5	129.1 ± 16.8	< 0.001
DBP, mmHg	77.5 ± 10.4	77.8 ± 10.2	77.6 ± 10.5	77.5 ± 10.4	76.9 ± 10.0	0.184
Current smoker, n (%)	5963 (57.2)	860 (57.8)	2592 (58.4)	1909 (55.8)	602 (55.5)	0.075
Hypertension, n (%)	6725 (64.5)	865 (58.2)	2718 (61.2)	2397 (70)	745 (68.7)	< 0.001
Dyslipidemia, n (%)	7028 (67.4)	921 (61.9)	2848 (64.2)	2441 (71.3)	818 (75.4)	< 0.001
eGFR, ml/min	91.3 ± 15.0	94.5 ± 14.0	91.4 ± 14.3	90.3 ± 15.4	89.6 ± 16.7	< 0.001
LVEF, %	62.8 ± 7.3	62.8 ± 7.2	63.1 ± 7.1	62.4 ± 7.5	62.4 ± 7.4	< 0.001
TG, mmol/L	1.54 (1.15, 2.12)	1.47 (1.11, 1.97)	1.51 (1.12, 2.06)	1.61 (1.21, 2.25)	1.57 (1.16, 2.17)	< 0.001
TC, mmol/L	4.05 (3.44, 4.81)	3.99 (3.37, 4.69)	4.09 (3.47, 4.82)	4.04 (3.44, 4.87)	4.02 (3.39, 4.80)	< 0.001
HDL-C, mmol/L	0.99 (0.84, 1.17)	0.98 (0.84, 1.19)	1.01 (0.85, 1.20)	0.98 (0.83, 1.15)	0.96 (0.81, 1.13)	< 0.001
LDL-C, mmol/L	2.35 (1.86, 3.01)	2.31 (1.79, 2.92)	2.39 (1.88, 3.04)	2.33 (1.86, 3.00)	2.31 (1.82, 2.96)	< 0.001
Lp (a), mg/L	183.79 (78.22, 410.74)	171.59 (74.97, 415.70)	190.58 (83.91, 418.06)	177.92 (71.85, 400.19)	181.61 (76.74, 402.38)	0.012
FPG, mmol/L	5.55 (4.96, 6.81)	5.00 (4.63, 5.46)	5.20 (4.82, 5.66)	6.62 (5.66, 8.05)	7.77 (6.31, 9.84)	< 0.001
HbA1c, %	6.2 (5.8, 7.0)	5.5 (5.3, 5.6)	6.0 (5.8, 6.2)	7.0 (6.6, 7.8)	8.1 (7.2, 9.0)	< 0.001
New-onset CAD, n (%)	6669 (63.9)	1026 (69)	2926 (65.9)	2096 (61.3)	621 (57.2)	< 0.001
Multi-vessel CAD, n (%)	7909 (75.8)	995 (66.9)	3251 (73.2)	2749 (80.3)	914 (84.2)	< 0.001
**Antidiabetic drugs, n (%)**						< 0.001
None	7871 (75.4)	1487 (100)	4439 (100)	1945 (56.8)	0 (0)	
OHA	1477 (14.2)	0 (0)	0 (0)	1477 (43.2)	0 (0)	
Insulin	1085 (10.4)	0 (0)	0 (0)	0 (0)	1085 (100)	
Antihypertensive drugs, n (%)	2358 (22.6)	229 (15.4)	863 (19.4)	942 (27.5)	324 (29.9)	< 0.001
Antiplatelet drugs, n (%)	4625 (44.3)	580 (39)	1902 (42.8)	1604 (46.9)	539 (49.7)	< 0.001
Antilipidemic drugs, n (%)	5218 (50.0)	517 (34.8)	1769 (39.9)	2087 (61)	845 (77.9)	< 0.001

BMI: body mass index; CAD: coronary artery disease; DBP: diastolic blood pressure; DM: diabetes mellitus; eGFR: estimated glomerular filtration rate; FPG: fasting plasma glucose; HbA1c: glycated hemoglobin; HDL-C: high-density lipoprotein cholesterol; IR: insulin resistance; LDL-C: low-density lipoprotein cholesterol; Lp(a): lipoprotein(a); LVEF: left ventricular ejection fraction; NGR: normal glucose regulation; OHA: oral hypoglycemic agents; pre-DM: prediabetes; Rx: prescription; SBP: systolic blood pressure; TC: total cholesterol; TG: triglycerides; TyG: triglyceride-glucose

**Fig. 2:**
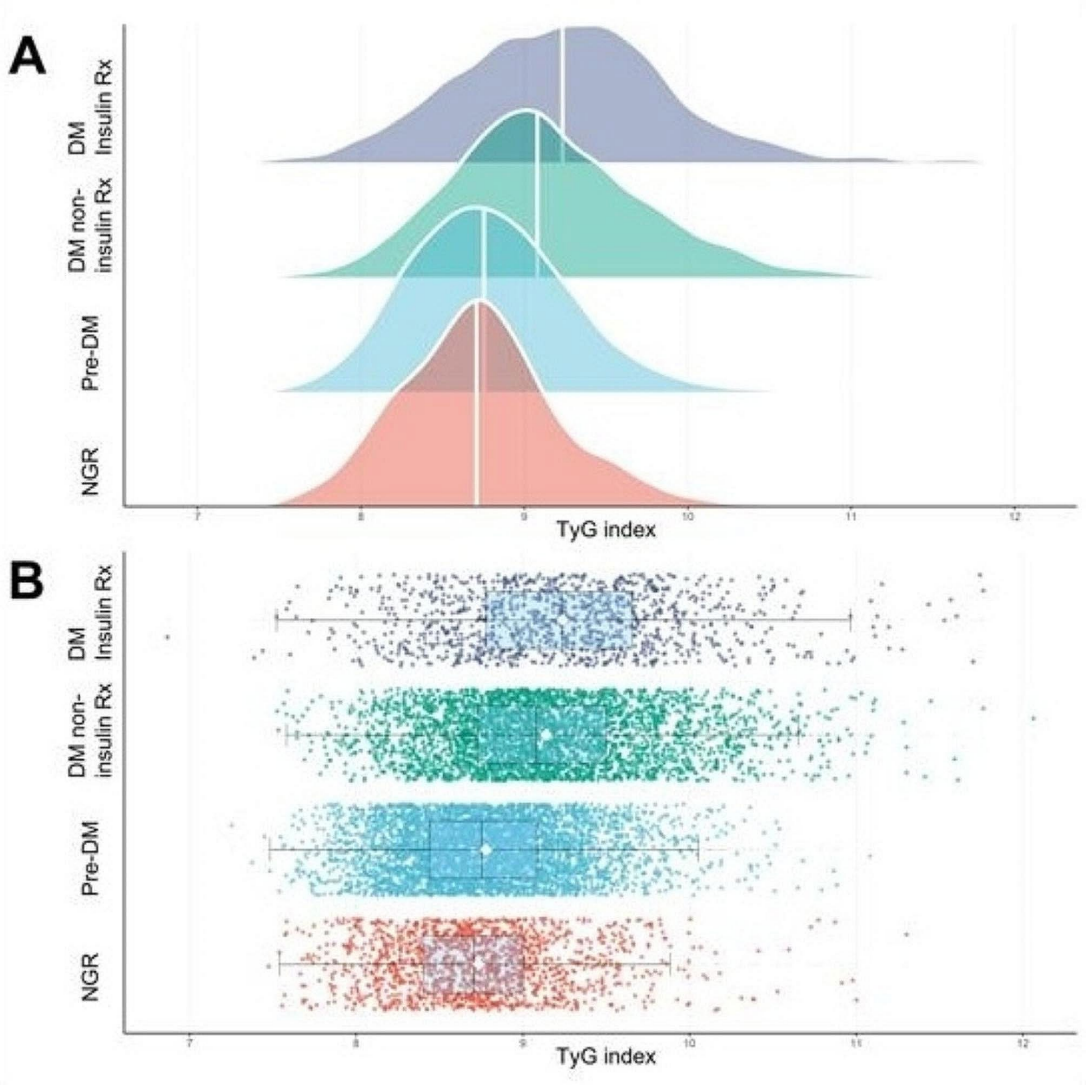
Distribution of TyG index across glucose metabolism subgroups. Panel A: Ridge plots display the density of TyG index across glucose metabolism subgroups, with the central white vertical line indicating the median value. Panel B: Data points represent individual patients, and box plots display median (central vertical line), mean (central white diamond), IQR (vertical lines on box edges), and values no further than 1.5 times the IQR from the edges of the box (whiskers and outer vertical lines). Abbreviations: DM: diabetes mellitus; NGR: normal glucose regulation; pre-DM: prediabetes; Rx: prescription; TyG: triglyceride-glucose

The distributions of TyG index levels stratified by glucose metabolism states are presented in Fig. 2. The mean levels of TyG index were 8.74 ± 0.51, 8.78 ± 0.49, 9.14 ± 0.61, and 9.24 ± 0.68 in NGR, pre-DM, DM non-insulin Rx, and DM insulin Rx groups, respectively. Density curves showed that participants with more severe abnormal glucose metabolism state or insulin use tended to have higher TyG index levels compared to those with NGR, and statistical differences were observed among them (Table 3).

### Associations between the TyG index and CAD severity

**Fig. 3:**
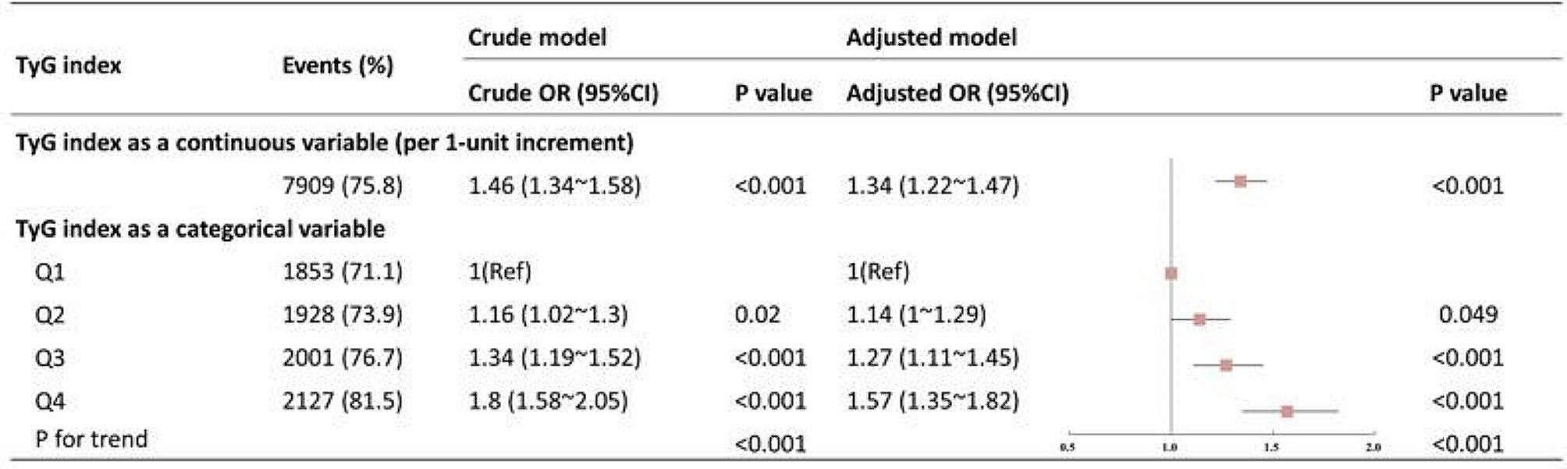
Association between TyG index and multi-vessel CAD in total population**. **Adjusted model: adjusted for age, sex, BMI, SBP, current smoking status, hypertension, dyslipidemia, eGFR, LVEF, HDL-C, LDL-C, Lp(a), antidiabetic drugs, antihypertensive drugs, antiplatelet drugs, and antilipidemic drugs. Abbreviations: CI: confidence intervals; DM: diabetes mellitus; NGR: normal glucose regulation; OR: odds ratio; pre-DM: prediabetes; Rx: prescription; TyG: triglyceride-glucose

The associations between the TyG index and CAD severity in the overall population are illustrated in Fig. 3. Irrespective of adjustment for confounding factors, the TyG index exhibited a significant positive correlation with multi-vessel CAD. Both crude (OR: 1.46, 95% CI: 1.34–1.58 per 1-unit increment) and adjusted (OR: 1.34, 95% CI: 1.22–1.47 per 1-unit increment) odds ratios were calculated. The TyG index was divided into quartiles, using the Q1 group as a reference to assess its relationship with multi-vessel CAD. With increasing TyG quartiles, the rate of multi-vessel CAD gradually rises, escalating from 71.1% in Q1 to 81.5% in Q4. After adjusting for various confounding factors including age, sex, BMI, SBP, current smoking status, hypertension, dyslipidemia, eGFR, LVEF, HDL-C, LDL-C, Lp(a), and current medication with antidiabetic, antihypertensive, antiplatelet, and antilipidemic drugs, the odds of multi-vessel CAD increased linearly across Q2 (OR: 1.14; 95% CI: 1.00–1.29), Q3 (OR: 1.24; 95% CI: 1.11–1.45), and Q4 (OR: 1.57; 95% CI: 1.35–1.82) groups compared to the Q1 reference group. The P-value for trend was < 0.001, indicating a linear positive correlation between the TyG index and multi-vessel CAD.

**Fig. 4:**
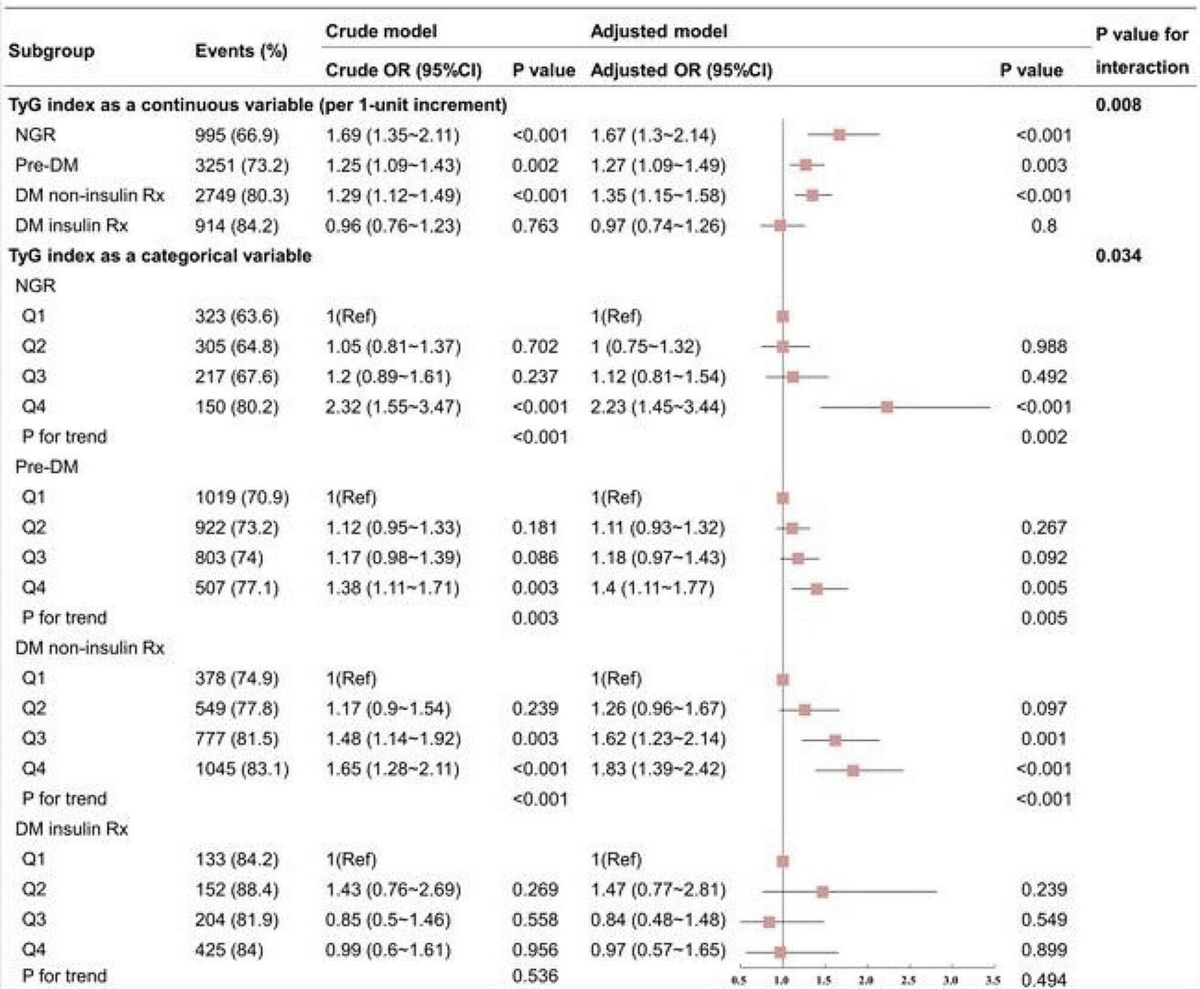
Glucose metabolism subgroup and interaction analysis of multi-vessel CAD. Adjusted model: adjusted for age, sex, BMI, SBP, current smoking status, hypertension, dyslipidemia, eGFR, LVEF, HDL-C, LDL-C, Lp(a), antihypertensive drugs, antiplatelet drugs, and antilipidemic drugs. Abbreviations: CI: confidence intervals; DM: diabetes mellitus; NGR: normal glucose regulation; OR: odds ratio; pre-DM: prediabetes; Rx: prescription; TyG: triglyceride-glucose

Figure 4 shows the relationship between the TyG index and CAD severity across various glucose metabolism states. With the increase in the TyG index in both crude and adjusted models, the risks of multi-vessel CAD among the NGR, pre-DM, and DM non-insulin Rx groups are more significant than that among DM insulin Rx group (P for interaction = 0.008). The adjusted ORs of multi-vessel CAD in the NGR (OR: 1.67; 95% CI: 1.3–2.14), pre-DM (OR: 1.27; 95% CI: 1.09–1.49), and DM non-insulin Rx (OR: 1.35; 95% CI: 1.15–1.58) groups were statistically significant. Additionally, the TyG index in the DM insulin Rx group showed no association with multi-vessel CAD in either crude or adjusted models (*P* = 0.8). Likewise, in addition to the TyG index quartiles by glucose metabolism state subgroups, the influence of the TyG index as a categorical variable on multi-vessel CAD was analyzed. Compared with the Q1 reference group, the ORs of multi-vessel CAD in the Q2, Q3, and Q4 groups exhibited a consistent upward trend across the NGR, pre-DM, and DM non-insulin Rx subgroups, with a P for trend < 0.01 in each subgroup. However, such a trend was absent in the DM insulin Rx subgroup (P for trend = 0.494), with a significant difference from the three subgroups (P for interaction = 0.034).

**Fig. 5:**
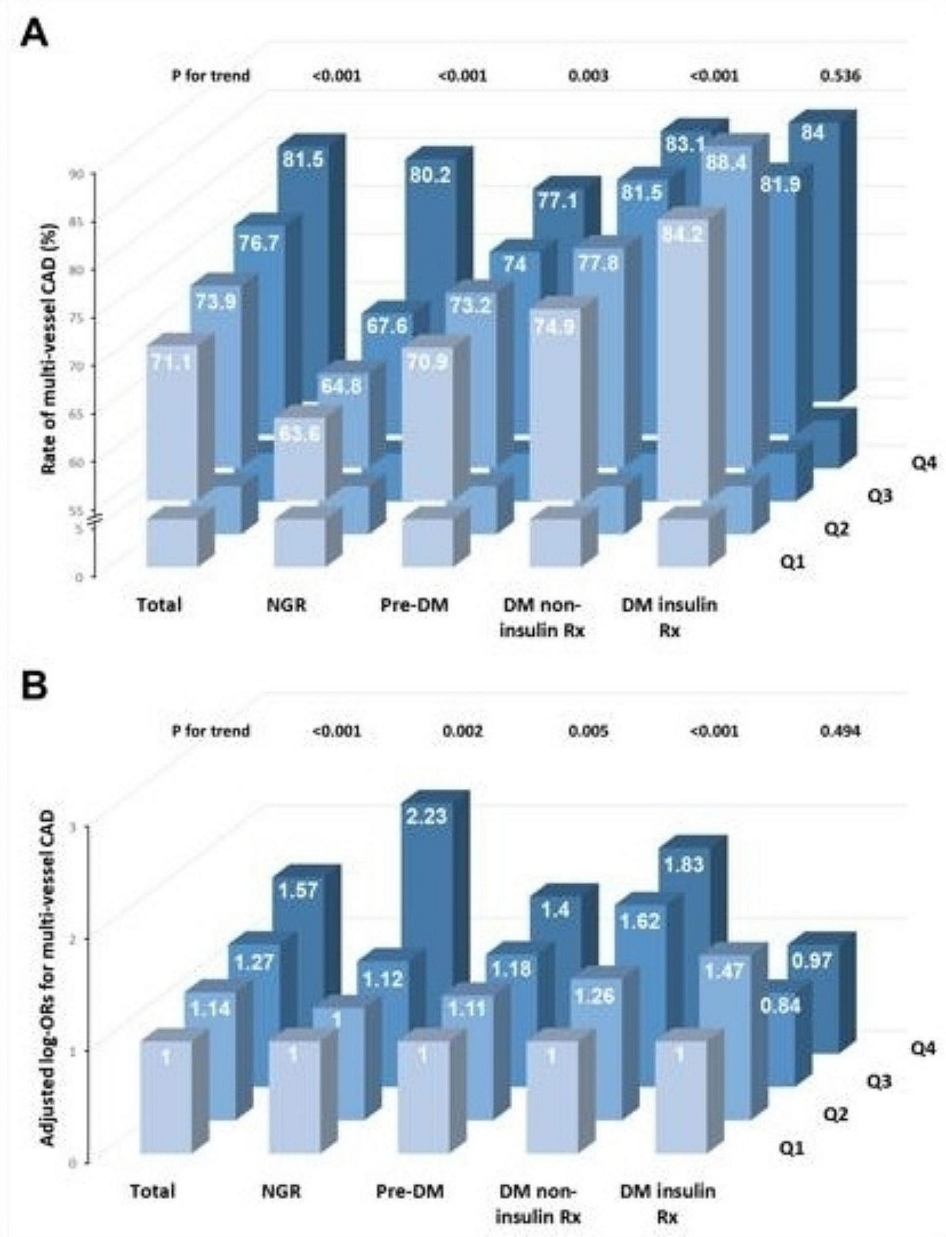
Rates and ORs of multi-vessel CAD according to TyG levels across different glucose metabolism states. Panel A: Rates of multi-vessel CAD; Panel B: Adjusted log-ORs for multi-vessel CAD. Adjusted for age, sex, BMI, SBP, current smoking status, hypertension, dyslipidemia, eGFR, LVEF, HDL-C, LDL-C, Lp(a), antidiabetic drugs (only for the total population), antihypertensive drugs, antiplatelet drugs, and antilipidemic drugs. Abbreviations: DM: diabetes mellitus; NGR: normal glucose regulation; OR: odds ratio; pre-DM: prediabetes; Rx: prescription; TyG: triglyceride-glucose

As summarized in Fig. 5, in the overall population, both the rate and adjusted OR of multi-vessel CAD escalate with elevated TyG quartiles. This trend remains consistent in the NGR, pre-DM, and DM non-insulin Rx groups. However, in the DM insulin Rx group, TyG shows no significant association with either the rate or adjusted OR of multi-vessel CAD.

### RCS analysis investigating the relationship between the TyG index and CAD severity

**Fig. 6:**
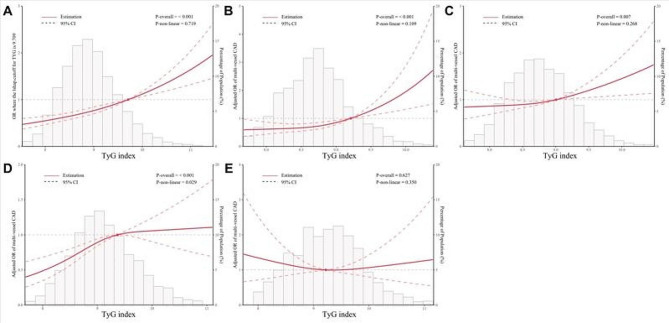
Restricted cubic splines demonstrating adjusted OR of multi-vessel CAD associated with TyG index across total population and glucose metabolism subgroups. Panel A: Total population; Panel B: NGR; Panel C: Pre-DM; Panel D: DM non-insulin Rx; Panel E: DM insulin Rx. Adjusted for age, sex, BMI, SBP, current smoking status, hypertension, dyslipidemia, eGFR, LVEF, HDL-C, LDL-C, Lp(a), antidiabetic drugs (only for the total population), antihypertensive drugs, antiplatelet drugs, and antilipidemic drugs. Abbreviations: CI: confidence intervals; OR: odds ratio; TyG: triglyceride-glucose

Figure 6 employs RCS to flexibly model and visualize the relationship between the TyG index and multi-vessel CAD. After adjusting for all mentioned covariates in the adjusted model, we observed linear associations between the TyG index and multi-vessel CAD in the overall population (P-overall < 0.001, P-nonlinear = 0.719). Similarly, the TyG index exhibited linear correlations with multi-vessel CAD in both the NGR and pre-DM populations (P-overall < 0.01 for both, P-nonlinear > 0.05 for both). However, the TyG index exhibited nonlinear associations in the DM non-insulin Rx population (P-overall < 0.001 and P-nonlinear = 0.029), indicating a threshold saturation effect on multi-vessel CAD when the TyG index exceeded 9.37. Similarly, in line with the logistic regression results, the TyG index exhibited neither linear nor nonlinear associations with multi-vessel CAD in the DM insulin Rx population (P-overall = 0.627, P-nonlinear = 0.350).

### Mediation analysis of the TyG index with CAD severity

**Fig. 7:**
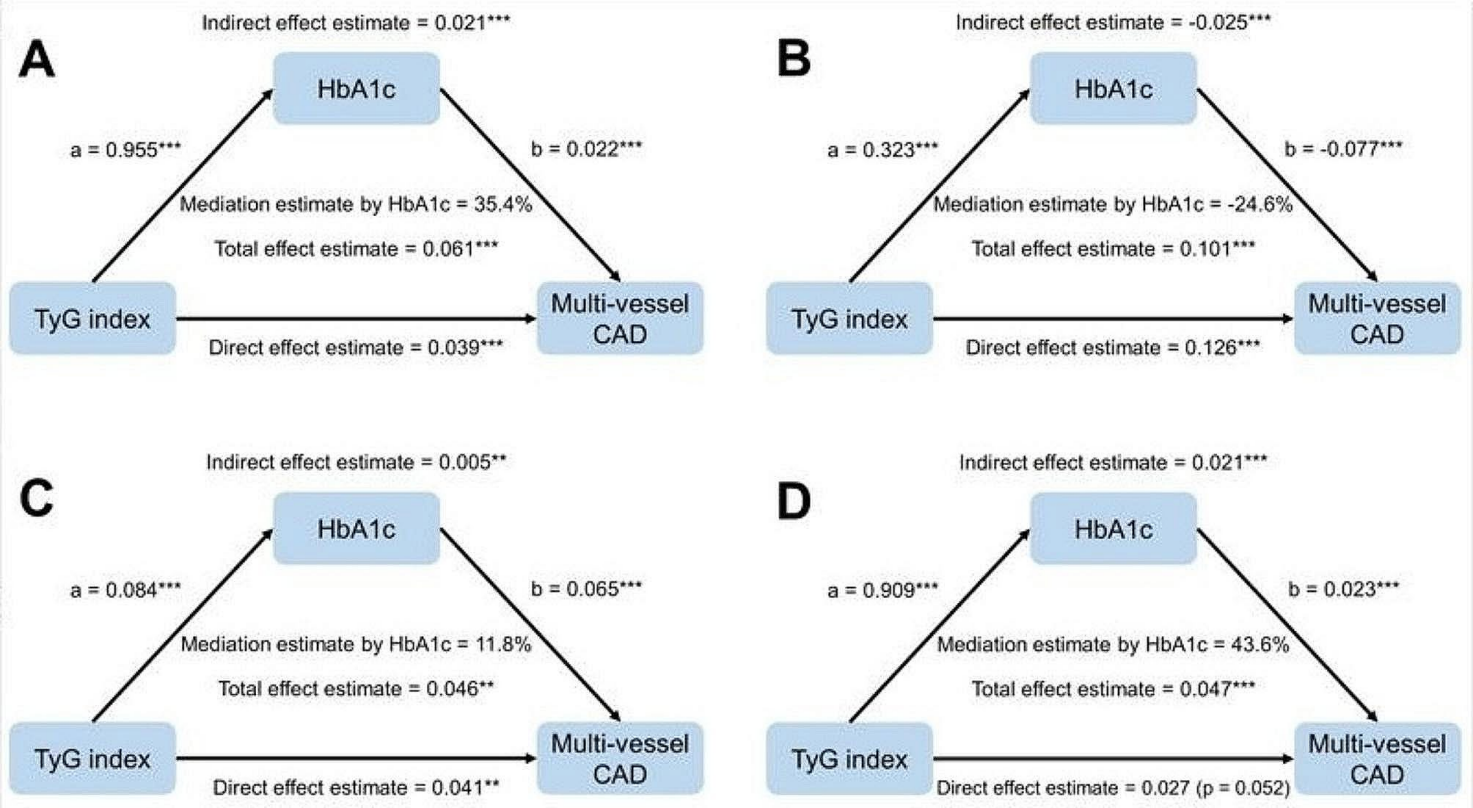
Mediation analysis on the association of TyG index with multi-vessel CAD across total population and glucose metabolism subgroups. Panel A: total population; Panel B: NGR group; Panel C: pre-DM group; Panel D: DM non-insulin Rx group. Adjusted for age, sex, BMI, SBP, current smoking status, hypertension, dyslipidemia, eGFR, LVEF, HDL-C, LDL-C, Lp(a), antidiabetic drugs (only for the total population), antihypertensive drugs, antiplatelet drugs, and antilipidemic drugs. * *p* < 0.05. ** *p* < 0.01. *** *p* < 0.001. Abbreviations: CAD: coronary artery disease; HbA1c: hemoglobin A1c; TyG: triglyceride-glucose

Mediation analyses indicated that HbA1c partially mediated the association between the TyG index and multi-vessel CAD (Fig. 7). In the entire population and three subgroups (NGR, pre-DM, and DM non-insulin Rx), the total effect, indirect effect, and direct effect were were statistically significant, except for the direct effect observed in the DM non-insulin Rx subgroup (*P* = 0.052). In the entire population, the proportion of indirect effects of HbA1c-mediated multi-vessel CAD were 35.4%. Within the NGR, pre-DM, and DM non-insulin Rx subgroups, the proportions of indirect effects mediated by HbA1c on multi-vessel CAD were − 24.6%, 11.8%, and 43.6%, respectively.

### Risk prediction performance of the TyG index for multi-vessel CAD

Table 4 presents the added predictive ability and reclassification statistics of the triglyceride-glucose index for multi-vessel CAD. In the entire population, the prediction model for multi-vessel CAD, incorporating established risk factors, achieved a C-statistic value of 0.64. Incorporating the TyG index into this model led to significant enhancements in C-statistic, NRI, and IDI (all P-values < 0.01). Across the glucose metabolism subgroups, the C-statistic values of the established model were 0.661, 0.627, 0.615, and 0.621 for patients with NGR, pre-DM, DM non-insulin Rx, and DM insulin Rx, respectively. The addition of the TyG index to the established model led to improved C-statistic, NRI, and IDI (all P-values < 0.05) in the NGR subgroup, enhanced NRI and IDI in the DM non-insulin Rx subgroup (all P-values < 0.001), improved NRI in the pre-DM subgroup (P-value = 0.003), while no significant improvement in C-statistic, NRI, or IDI was observed in the DM insulin Rx subgroup.

**Table 4:** Improvement in discrimination and risk reclassification for multi-vessel CAD after adding TyG index

	C-Statistic (95% CI)	*P* value	NRI (95% CI)	*P* value	IDI (95% CI)	*P* value
**Total population**						
Established model	0.640 (0.628, 0.653)	Reference	Reference		Reference	
Established model + TyG index	0.645 (0.633, 0.658)	0.005	0.118 (0.073, 0.162)	< 0.001	0.004 (0.003, 0.005)	< 0.001
**NGR**						
Established model	0.661 (0.631, 0.691)	Reference	Reference		Reference	
Established model + TyG index	0.673 (0.644, 0.703)	0.04	0.165 (0.058, 0.272)	0.003	0.011 (0.005, 0.016)	< 0.001
**Pre-DM**						
Established model	0.627 (0.609, 0.645)	Reference	Reference		Reference	
Established model + TyG index	0.630 (0.612, 0.648)	0.231	0.059 (0.006, 0.126)	0.077	0.002 (7e-04, 0.003)	0.003
**DM non-insulin Rx**						
Established model	0.615 (0.591, 0.639)	Reference	Reference		Reference	
Established model + TyG index	0.621 (0.598, 0.645)	0.191	0.154 (0.071, 0.237)	< 0.001	0.004 (0.002, 0.006)	< 0.001
**DM insulin Rx**						
Established model	0.621 (0.577, 0.665)	Reference	Reference		Reference	
Established model + TyG index	0.621 (0.577, 0.665)	0.854	0.053 (-0.111, 0.216)	0.528	0 (-0.001, 0.001)	0.838

The established model includes the following variables: age, sex, BMI, SBP, current smoking status, hypertension, dyslipidemia, eGFR, LVEF, HDL-C, LDL-C, Lp(a), antidiabetic drugs, antihypertensive drugs, antiplatelet drugs, and antilipidemic drugs. Abbreviations: CAD: coronary artery disease; CI: confidence intervals; DM: diabetes mellitus; IDI: integrated discrimination improvement; NGR: normal glucose regulation; NRI: net reclassification improvement; pre-DM: prediabetes; Rx: prescription; TyG: triglyceride-glucose

### Sensitivity analysis

**Fig. 8:**
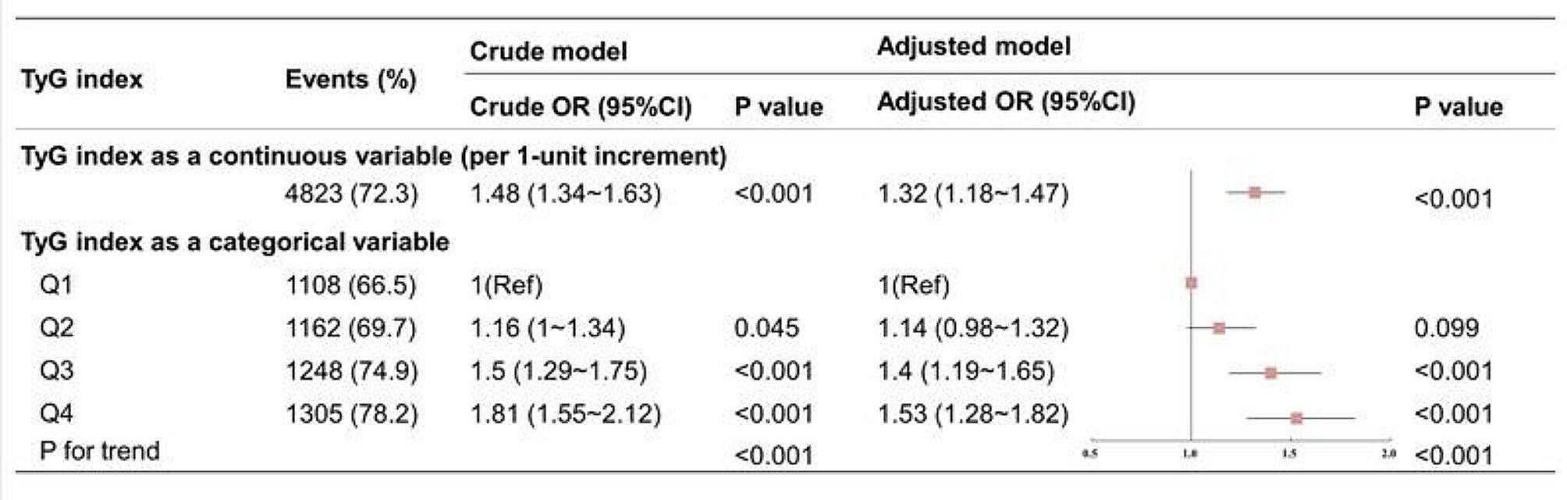
Association between TyG index and multi-vessel CAD in new-onset CAD population. Adjusted model: adjusted for age, sex, BMI, SBP, current smoking status, hypertension, dyslipidemia, eGFR, LVEF, HDL-C, LDL-C, Lp(a), antidiabetic drugs, antihypertensive drugs, antiplatelet drugs, and antilipidemic drugs. Abbreviations: CI: confidence intervals; OR: odds ratio; TyG: triglyceride-glucose

**Fig. 9:**
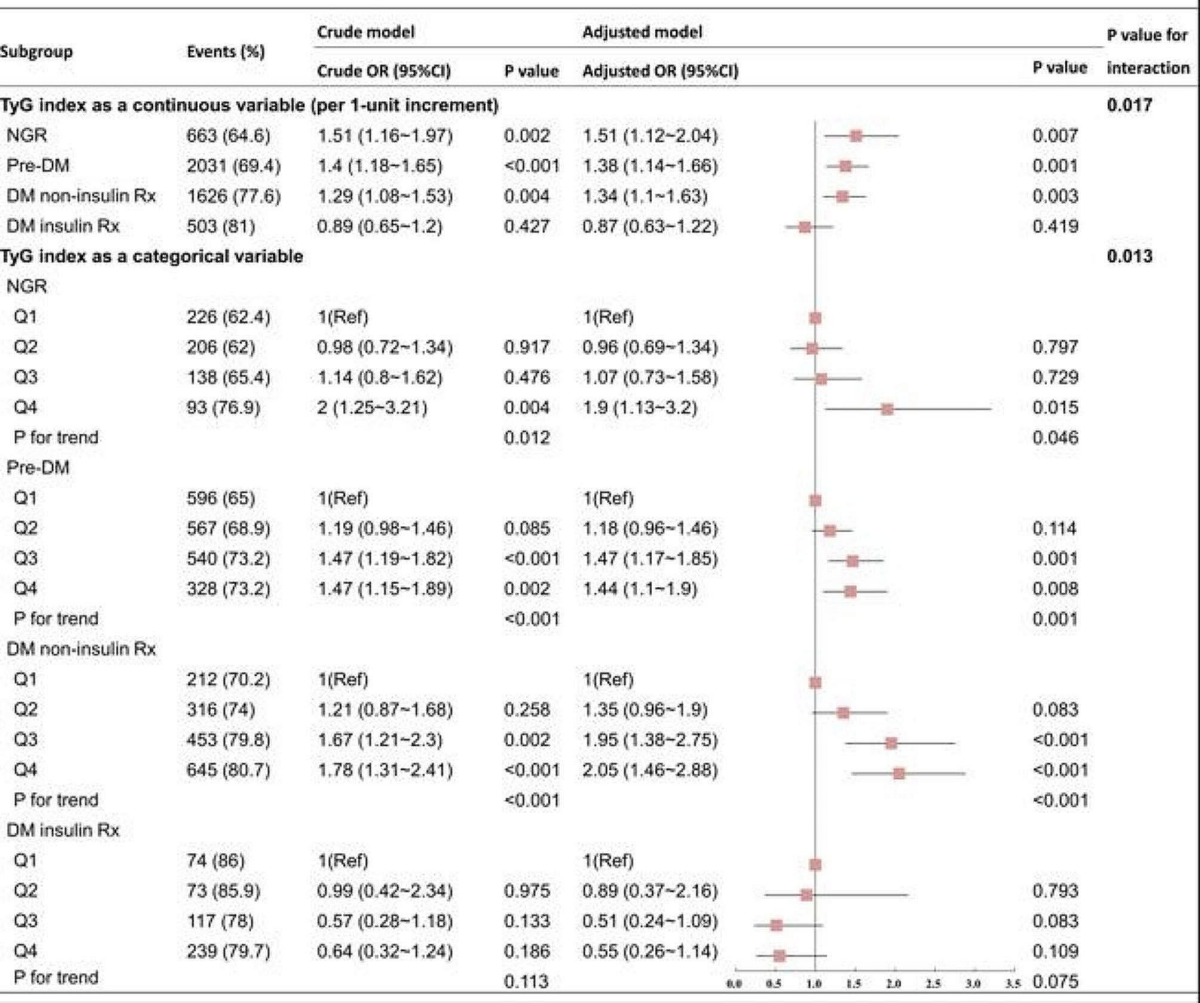
Glucose metabolism subgroup and interaction analysis of multi-vessel CAD in new-onset CAD population. Adjusted model: adjusted for age, sex, BMI, SBP, current smoking status, hypertension, dyslipidemia, eGFR, LVEF, HDL-C, LDL-C, Lp(a), antihypertensive drugs, antiplatelet drugs, and antilipidemic drugs. Abbreviations: CAD: coronary artery disease; CI: confidence intervals; DM: diabetes mellitus; NGR: normal glucose regulation; OR: odds ratio; pre-DM: prediabetes; Rx: prescription; TyG: triglyceride-glucose

**Fig. 10:**
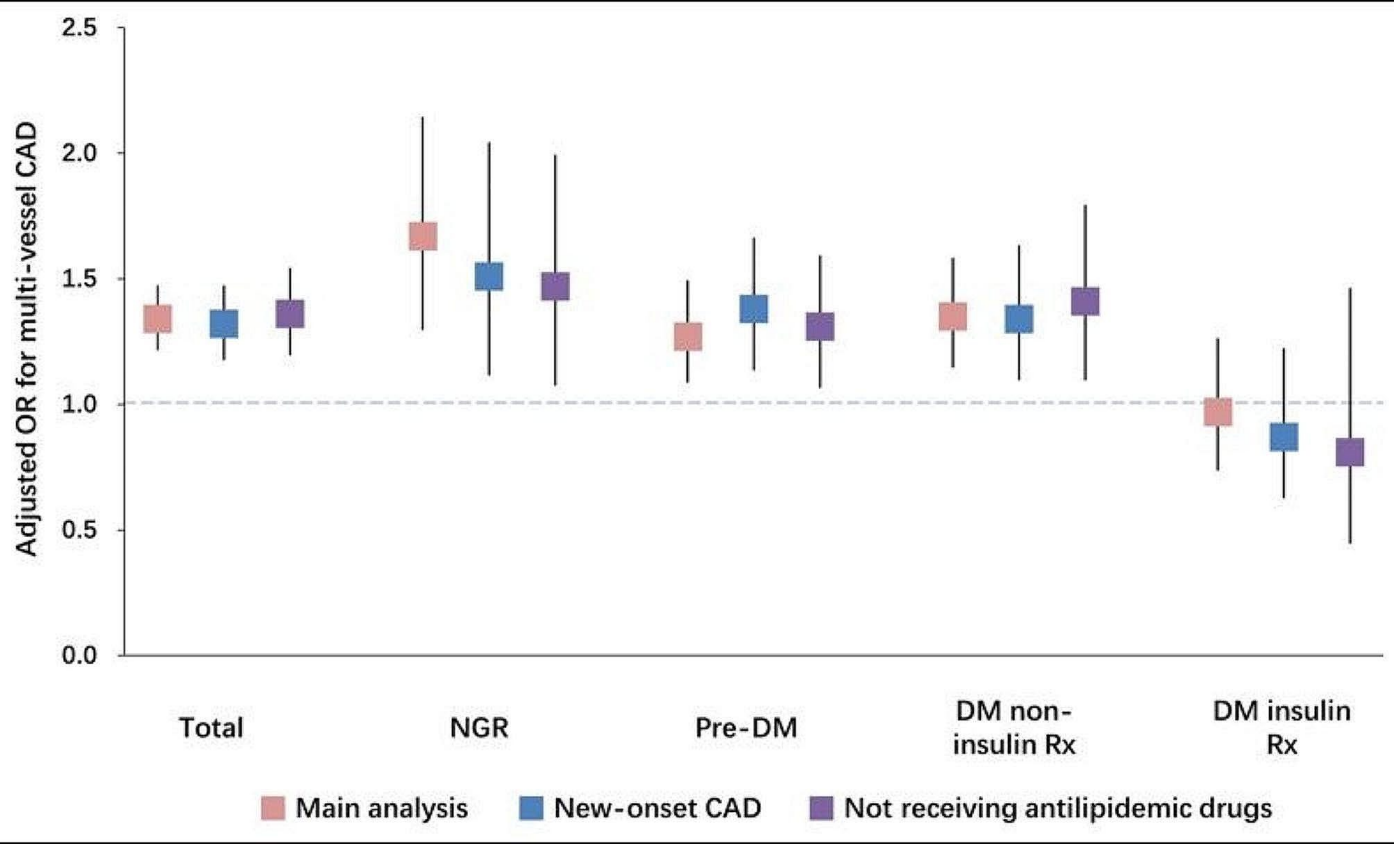
OR Comparison Plot. OR comparison plot among different statistical models: main analysis, sensitivity analysis in the new-onset CAD population, and sensitivity analysis in the population not receiving antilipidemic drugs. Abbreviations: CAD: coronary artery disease; DM: diabetes mellitus; NGR: normal glucose regulation; OR: odds ratio; pre-DM: prediabetes; Rx: prescription; TyG: triglyceride-glucose

Two sensitivity analyses were performed. Firstly, individuals with pre-existing CAD were excluded from a sensitivity analysis to address conflicting conclusions in previous studies involving new-onset CAD populations (Additional file 1: Table S1). The new-onset case analysis consistently demonstrated significant associations between the TyG index and multi-vessel CAD in both the entire new-onset CAD population and various glucose metabolism subgroups (Figs. 8 and 9). Secondly, to mitigate the potential influence of antilipidemic drugs on TG and TyG, 5218 patients using these medications were excluded. This exclusion had no significant impact on our results (Additional file 1: Table S2, Fig. S1-S2). The summarized results of the primary analysis and sensitivity analyses are presented in Fig. 10.

## Discussion

In our study, we conducted a comprehensive investigation into the relationship between the TyG index and CAD severity across different glucose metabolism states. Four main findings emerged from our investigation. Firstly, we addressed the intense controversy among previous relatively small-sample studies in this field. In the largest sample examined to date, we confirmed the independent association between the TyG index and multi-vessel CAD, regardless of patients’ glucose metabolism state (NGR, pre-DM, or DM), with the only exception when insulin therapy was administered. Secondly, a linear relationship between the TyG index and multi-vessel CAD was observed in the NGR and pre-DM populations, while a nonlinear relationship and threshold saturation effect were noted in the DM non-insulin Rx population. Thirdly, HbA1c exhibited varied mediating roles in the association between the TyG index and multi-vessel CAD, with its mediation becoming more prominent as glucose metabolism abnormalities worsened. Fourthly, the TyG index can provide moderate incremental predictive value for multi-vessel CAD beyond established risk factors, with the most notable effect observed in the NGR population.

Multi-vessel CAD is strongly associated with a poor prognosis in CAD and can increase the complexity of PCI procedures as well as subsequent medication therapy [3]. The exploration of its pathogenesis and predictive indicators has long been a focal point of research. The TyG index, representing IR, is strongly linked to the onset and progression of atherosclerotic cardiovascular diseases, particularly CAD [21]. The investigation into the relationship between TyG and multi-vessel CAD is also receiving increasing attention. Wu et al. found that a higher TyG index increased the risk of arterial stiffness [22]. Thai et al. first reported that an elevated TyG index identified patients at high risk of coronary artery stenoses, correlating with both the number and severity of stenoses [23]. Furthermore, Su et al., Zhang et al., Wang et al., and Xie et al. have successively validated the association between the TyG index and multi-vessel CAD in larger cohorts [16–18, 24]. However, two major controversies remain in this field: First, studies have differing conclusions on the relationship between the TyG index and multi-vessel CAD across glucose metabolism subgroups. For instance, Su et al. found this association in the DM group but not in the pre-DM or NGR groups, while Wang et al. found it in the pre-DM group but not in the DM or NGR groups. Second, contradicting the conclusions of the aforementioned studies, a recent study found no association between the TyG index and multi-vessel CAD in new-onset CAD patients, regardless of glucose metabolism state [15]. The authors attribute this conflicting result to the selection of enrolled patients and Neyman bias in previous studies, but their study also suffers from a small sample size. These controversies confound our understanding of the relationship between the TyG index and multi-vessel CAD, necessitating larger and more comprehensive studies to resolve these issues.

Compared to prior studies, our research offers several advantages: Firstly, it boasts the largest sample size, enhancing its persuasive capacity. Secondly, it includes a most extensive array of adjustment factors, encompassing various risk factors, laboratory parameters, and medication utilization. This encompasses novel metrics such as Lp(a), previously overlooked in research. Thirdly, considering the significant impact of insulin on IR, it distinguishes between insulin and common antidiabetic medications, and analyzes them separately by grouping. Fourthly, it performs comprehensive sensitivity analyses in cohorts with potential controversies, including individuals with newly diagnosed CAD and those not prescribed antilipidemic drugs. Drawing from these aforementioned advantages, our study elucidates the association between the TyG index and multi-vessel CAD across the population and within different glucose metabolism subgroups. This finding aligns with the main conclusions drawn from the majority of prior research and meta-analyses [13, 16–18, 23, 25]. Contradictory outcomes noted in certain previous studies across different glucose metabolism subgroups could stem from their relatively limited sample sizes or inadequate adjustment for insulin [16, 18]. Moreover, sensitivity analysis results bolster the resilience of findings in the new-onset CAD cohort, indicating that discrepant conclusions in a recent study could be attributed to its smaller sample size (6669 cases vs. 431 cases) [15].

Importantly, given the intricate interplay among IR, glucose metabolism status, and blood glucose levels, we observed significant variations in the dose-response relationship between the TyG index and CAD severity across different glucose metabolism subgroups. Figure 5 shows that the association between the TyG index and CAD severity is significantly stronger in the NGR group, especially in the 4th quartile, compared to other groups. This association largely explains the overall relationship observed in the total population. This finding is plausible, since elevated glucose levels in other groups may interfere with the TyG index’s association with CAD severity. This interference is most pronounced in the DM insulin Rx group, where glucose variability may diminish this association. Furthermore, Fig. 6 indicates that the increased OR is primarily driven by subjects in the top 25th percentile of the NGR group, while the pre-DM group shows this increase only in the top 50th percentile. Conversely, in the DM non-insulin Rx group, the increase is confined to the bottom 50th percentile. Notably, in the DM insulin Rx group, the TyG index demonstrates a nearly U-shaped relationship. When aggregating all subjects, the data suggest a somewhat linear relationship, which is an artifact of differing behaviors across the various groups. Additionally, the confidence intervals for these curves are relatively wide, indicating variability in the data through different subgroups. Given the dose-response differences in TyG index and CAD severity across different glucose metabolism subgroups, the interpretation of the TyG index’s relationship with CAD severity must consider glucose metabolism states [16, 18].

In addition to addressing major controversies in this field, another significant finding of our study is the notable interaction effect of insulin treatment. Previously, it was believed that the TyG index was developed as a reliable biochemical surrogate for identifying IR in both diabetic and non-diabetic individuals [12]. Unlike other IR indices such as the homeostasis model assessment of insulin resistance (HOMA-IR), quantitative insulin sensitivity check index (QUICKI), and homeostasis model assessment of β-cell function (HOMA-β), the TyG index does not require insulin quantification [26]. Therefore, it has traditionally been considered less influenced by insulin treatment [12, 26, 27]. Our study found that the TyG index levels progressively increased across the NGR, pre-DM, DM non-insulin Rx, and DM insulin Rx groups. From the perspective of the components of the TyG index, blood glucose and TG by definition increase with worsening blood glucose control. This is also consistent with the understanding that greater glucose metabolism abnormalities correspond to more severe IR. However, within the DM insulin Rx group, the positive correlation between TyG and CAD severity was not established, and this difference exhibited a significant interaction effect. This suggests that the TyG index may not be entirely unaffected by exogenous insulin. We hypothesize that the interaction effect related to exogenous insulin may occur through the following pathways: First, high blood glucose can induce insulin resistance by acting on post-receptor signaling pathways, and moderate exogenous insulin supplementation can effectively lower blood glucose levels, thereby reducing the fasting glucose component of the TyG index and improving insulin sensitivity [28]. Second, moderate exogenous insulin can promote the conversion of fatty acids into triglycerides in adipose tissue, reducing free fatty acids in the blood and subsequently lowering plasma triglyceride levels [29]. Third, persistent hyperinsulinemia itself can induce central and peripheral insulin resistance; long-term or inappropriate use of high doses of insulin may lead to weight gain and fat accumulation, which can, in turn, increase IR and elevate the TyG index [30, 31]. Fourth, excessive and inappropriate use of insulin, which may result in hypoglycemia, can further induce insulin resistance by stimulating the secretion of counter-regulatory hormones, mainly glucagon, along with catecholamines, cortisol, and growth hormone [32, 33]. Therefore, the impact of insulin therapy on the TyG index and IR depends on the overall net effect, which is associated with factors such as disease duration, degree of hyperglycemia, and duration of treatment. This dual role of insulin may explain the observed interaction effect, at least regarding the correlation between the TyG index and multi-vessel CAD. Future studies may also need to prudently take into account the potential influence of exogenous insulin on the TyG index.

Furthermore, as the precise mechanism underlying the association between the TyG index and CAD remains incompletely understood, we conducted exploratory analyses through mediation effects in different glucose metabolism groups. The TyG index is currently recognized as a reliable indicator of IR, which could potentially account for this correlation [26, 34]. IR can induce glucose metabolism imbalance, contributing to hyperglycemia, which in turn triggers inflammation and oxidative stress. The molecular pathways linking IR and CAD encompass metabolic adaptability, endothelial dysfunction, abnormalities in coagulation, and dysfunction of smooth muscle cells [14, 31, 35–38]. A key discovery of our study is that HbA1c serves as a partial mediator in the relationship between the TyG index and multi-vessel CAD. HbA1c is created when hemoglobin from red blood cells binds with glucose. It represents the average blood glucose level over the preceding three months. Previous studies have suggested a link between increased HbA1c levels and the incidence and mortality of cardiovascular diseases [39–42]. Similarly, HbA1c is distinctly associated with CAD severity, including in both DM and non-DM patients, where higher HbA1c levels correlate with an increased risk of multi-vessel CAD [43–47]. Our study found that as glucose metabolism abnormalities worsen (from pre-DM to DM), the mediating effect proportion of HbA1c gradually increases (from 11.8 to 43.6%), indicating the importance of improving insulin resistance and glycemic control in both pre-DM and DM populations. Meanwhile, in the NGR population, we observed that the indirect effects of HbA1c-mediated associations between TyG and multi-vessel CAD were − 24.6%. On one hand, this suggests that HbA1c within the normal range has a protective effect on blood vessels, consistent with previous research findings: oscillating glucose can have detrimental effects on endothelial function and oxidative stress, and recurrent low blood glucose also contributes to CAD progression [48, 49]. On the other hand, this also suggests that in the NGR population, IR may primarily lead to coronary artery damage and multi-vessel CAD through the aforementioned non-glycemic effects [14, 34].

### Strengths and limitations

To the best of our knowledge, this study utilized the largest sample size to date for investigating the relationship between the TyG index and CAD severity, enhancing the reliability and generalizability of the findings. Through a large sample size and extensive subgroup analysis, this study effectively addressed the inconsistencies in previous research regarding different glucose metabolism subgroups and the presence or absence of new-onset coronary artery disease. However, our study has limitations. First, this study is based on a single-center Asian cohort, so extrapolating its conclusions to other racial groups requires further validation. Second, the data were obtained from a PCI cohort, which excluded patients with extremely low CAD complexity not requiring revascularization and those with extremely high CAD complexity undergoing CABG, potentially introducing bias. Third, lifestyle factors beyond smoking, such as diet, exercise, sleep, and stress, significantly impact IR [50]; however, similar to previous studies, our research was unable to fully adjust for these influences. Nevertheless, considering that lifestyle serves as an upstream determinant of IR, the lack of adjustment for these factors may not necessarily affect the conclusions drawn in this study. Fourth, this study is cross-sectional, limiting the ability to infer causality between the TyG index and CAD severity. Fifth, in this study, the incremental predictive value of the TyG index over traditional predictive factors is relatively small. Although there is a statistical difference, its specific clinical application scenarios should be comprehensively considered.

In summary, while this study provides robust and generalizable findings due to its large sample size and extensive subgroup analysis, future research should focus on multi-center, multi-ethnic cohort studies to enhance the generalizability of results, incorporate comprehensive lifestyle factor adjustments, and employ longitudinal designs to establish causal relationships between the TyG index and CAD severity.

## Conclusions

The TyG index was positively associated with CAD severity across all glucose metabolism states, except in individuals receiving insulin treatment. The TyG index and CAD severity show distinct dose-response relationships across different glucose metabolism subgroups. It might serve as a supplementary noninvasive predictor of CAD severity in addition to established factors, especially in NGR patients. HbA1c showed varying mediating effects on the relationship between the TyG index and CAD severity across different metabolic states. Further clinical and mechanistic studies are needed in the future.

### Electronic supplementary material

Below is the link to the electronic supplementary material.

Supplementary Material 1

## Data Availability

The datasets used in the study are available from the corresponding author upon reasonable request.

## Abbreviations

ADA: American Diabetes Association
BMI: Body mass index
CAG: Coronary artery angiography
CAD: Coronary artery disease
CI: Confidence intervals
DBP: Diastolic blood pressure
DM: Diabetes mellitus
eGFR: Estimated glomerular filtration rate
FPG: Fasting plasma glucose
HbA1c: Hemoglobin A1c
HDL-C: High-density lipoprotein cholesterol
HOMA-β: Homeostasis model assessment of β-cell function
HOMA-IR: Homeostasis model assessment of insulin resistance
IDI: Integrated discrimination improvement
IQR: Interquartile range
IR: Insulin resistance
LVEF: Left ventricular ejection fraction
LDL-C: Low-density lipoprotein cholesterol
Lp(a): Lipoprotein(a)
MACEs: Major adverse cardiovascular events
NGR: Normal glucose regulation
NRI: Net reclassification improvement
OHA: Oral hypoglycemic agents
OR: Odds ratio
PCI: Percutaneous coronary intervention
pre-DM: Prediabetes
QUICKI: Quantitative insulin sensitivity check index
RCS: Restricted cubic spline
ROC: Receiver operating characteristic
Rx: Prescription
SBP: Systolic blood pressure
SD: Standard deviation
TC: Total cholesterol
TG: Triglycerides
TyG: Triglyceride-glucose
