# Supplementary material for: Comprehensive analysis of the association between triglyceride-glucose index and coronary artery disease severity across different glucose metabolism states: a large-scale cross-sectional study from an Asian cohort
Source: Cardiovasc Diabetol. 2024 Jul 13;23:251. doi: 10.1186/s12933-024-02355-3 (PMC11245858; doi:10.1186/s12933-024-02355-3)
Supplement: Supplementary file 1 — Supplementary Material 1 [file 12933_2024_2355_MOESM1_ESM.pdf]

Table S1 Baseline characteristics according to new-onset and pre-existing CAD

Variables	Total (n = 10433)	New-onset CAD (n = 6669)	Pre-existing CAD (n = 3764)	P value
TYG index	8.89 (8.54, 9.28)	8.88 (8.54, 9.27)	8.89 (8.54, 9.29)	0.404
Age, year	58.3 ± 10.3	58.1 ± 10.2	58.7 ± 10.3	< 0.001
Male, n (%)	8053 (77.2)	4950 (74.2)	3103 (82.4)	< 0.001
BMI, kg/m^2	25.9 ± 3.2	25.9 ± 3.2	26.0 ± 3.1	0.021
SBP, mmHg	127.1 ± 16.5	127.7 ± 16.6	125.9 ± 16.1	< 0.001
DBP, mmHg	77.5 ± 10.4	77.8 ± 10.6	77.0 ± 10.1	< 0.001
Current smoker, n (%)	5963 (57.2)	3614 (54.2)	2349 (62.4)	< 0.001
Hypertension, n (%)	6725 (64.5)	4297 (64.4)	2428 (64.5)	0.917
Dyslipidemia, n (%)	7028 (67.4)	4326 (64.9)	2702 (71.8)	< 0.001
Glucose metabolism state, n (%)				< 0.001
NGR	1487 (14.3)	1026 (15.4)	461 (12.2)	
Pre-DM	4439 (42.5)	2926 (43.9)	1513 (40.2)	
DM non-insulin Rx	3422 (32.8)	2096 (31.4)	1326 (35.2)	
DM insulin Rx	1085 (10.4)	621 (9.3)	464 (12.3)	
eGFR, ml/min	91.3 ± 15.0	91.9 ± 14.4	90.3 ± 15.8	< 0.001
LVEF, %	62.8 ± 7.3	64.0 ± 6.3	60.5 ± 8.3	< 0.001
TG, mmol/L	1.54 (1.15, 2.12)	1.55 (1.15, 2.14)	1.52 (1.14, 2.08)	0.036
TC, mmol/L	4.05 (3.44, 4.81)	4.14 (3.52, 4.89)	3.90 (3.31, 4.65)	< 0.001
HDL-C, mmol/L	0.99 (0.84, 1.17)	1.00 (0.84, 1.19)	0.98 (0.82, 1.14)	< 0.001
LDL-C, mmol/L	2.35 (1.86, 3.01)	2.42 (1.91, 3.07)	2.24 (1.78, 2.88)	< 0.001
Lp (a), mg/L	183.79 (78.22, 410.74)	180.46 (77.68, 401.21)	188.80 (78.81, 427.83)	0.081
FPG, mmol/L	5.55 (4.96, 6.81)	5.51 (4.95, 6.66)	5.65 (4.97, 7.09)	< 0.001
HbA1c, %	6.2 (5.8, 7.0)	6.2 (5.8, 6.8)	6.3 (5.9, 7.2)	< 0.001
Multi-vessel CAD, n (%)	7909 (75.8)	4823 (72.3)	3086 (82)	< 0.001
Antidiabetic drugs, n (%)				< 0.001
None	7871 (75.4)	5124 (76.8)	2747 (73)	
OHA	1477 (14.2)	924 (13.9)	553 (14.7)	
Insulin	1085 (10.4)	621 (9.3)	464 (12.3)	
Antihypertensive drugs, n (%)	2358 (22.6)	214 (3.2)	2144 (57)	< 0.001
Antiplatelet drugs, n (%)	4625 (44.3)	1014 (15.2)	3611 (95.9)	< 0.001

Antilipidemic drugs, n (%)	5218 (50.0)	1559 (23.4)	3659 (97.2)	< 0.001

Abbreviations: BMI: body mass index; CAD: coronary artery disease; DBP: diastolic blood pressure; DM: diabetes mellitus; eGFR: estimated glomerular filtration rate; FPG: fasting plasma glucose; HbA1c: glycated hemoglobin; HDL-C: high-density lipoprotein cholesterol; IR: insulin resistance; LDL-C: low-density lipoprotein cholesterol; Lp(a): lipoprotein(a); LVEF: left ventricular ejection fraction; NGR: normal glucose regulation; OHA: oral hypoglycemic agents; pre-DM: prediabetes; Rx: prescription; SBP: systolic blood pressure; TC: total cholesterol; TG: triglycerides; TyG: triglyceride-glucose

Table S2. Baseline characteristics according to antilipidemic drug usage

**Variables**	**Total (n = 10433)**	**Antilipidemic drug usage**		
		**No (n = 5215)**	**Yes (n = 5218)**	**P value**
TYG index	8.89 (8.54, 9.28)	8.84 (8.51, 9.21)	8.93 (8.57, 9.36)	< 0.001
Age, year	58.3 ± 10.3	57.9 ± 10.4	58.7 ± 10.2	< 0.001
Male, n (%)	8053 (77.2)	3896 (74.7)	4157 (79.7)	< 0.001
BMI, kg/m^2	25.9 ± 3.2	25.8 ± 3.2	26.1 ± 3.1	< 0.001
SBP, mmHg	127.1 ± 16.5	127.5 ± 16.7	126.6 ± 16.3	0.006
DBP, mmHg	77.5 ± 10.4	77.9 ± 10.6	77.2 ± 10.2	< 0.001
Current smoker, n (%)	5963 (57.2)	2871 (55.1)	3092 (59.3)	< 0.001
Hypertension, n (%)	6725 (64.5)	3304 (63.4)	3421 (65.6)	0.019
Dyslipidemia, n (%)	7028 (67.4)	3269 (62.7)	3759 (72)	< 0.001
Glucose metabolism state, n (%)				< 0.001
NGR	1487 (14.3)	970 (18.6)	517 (9.9)	
Pre-DM	4439 (42.5)	2670 (51.2)	1769 (33.9)	
DM non-insulin Rx	3422 (32.8)	1335 (25.6)	2087 (40)	
DM insulin Rx	1085 (10.4)	240 (4.6)	845 (16.2)	
eGFR, ml/min	91.3 ± 15.0	91.9 ± 14.4	90.7 ± 15.4	< 0.001
LVEF, %	62.8 ± 7.3	64.0 ± 6.3	61.5 ± 7.9	< 0.001
TG, mmol/L	1.54 (1.15, 2.12)	1.55 (1.15, 2.14)	1.53 (1.15, 2.10)	0.540
TC, mmol/L	4.05 (3.44, 4.81)	4.14 (3.53, 4.90)	3.96 (3.35, 4.72)	< 0.001

HDL-C, mmol/L	0.99 (0.84, 1.17)	1.00 (0.85, 1.19)	0.98 (0.83, 1.16)	< 0.001
LDL-C, mmol/L	2.35 (1.86, 3.01)	2.42 (1.92, 3.09)	2.28 (1.80, 2.92)	< 0.001
Lp (a), mg/L	183.79 (78.22, 410.74)	183.33 (79.80, 405.78)	183.86 (76.72, 416.72)	0.995
FPG, mmol/L	5.55 (4.96, 6.81)	5.37 (4.91, 6.18)	5.84 (5.05, 7.49)	< 0.001
HbA1c, %	6.20 (5.80, 7.00)	6.10 (5.80, 6.60)	6.50 (6.00, 7.50)	< 0.001
New-onset CAD, n (%)	6669 (63.9)	5110 (98)	1559 (29.9)	< 0.001
Multi-vessel CAD, n (%)	7909 (75.8)	3696 (70.9)	4213 (80.7)	< 0.001
Antidiabetic drugs, n (%)				< 0.001
None	7871 (75.4)	4610 (88.4)	3261 (62.5)	
OHA	1477 (14.2)	365 (7)	1112 (21.3)	
Insulin	1085 (10.4)	240 (4.6)	845 (16.2)	
Antihypertensive drugs, n (%)	2358 (22.6)	88 (1.7)	2270 (43.5)	< 0.001
Antiplatelet drugs, n (%)	4625 (44.3)	805 (15.4)	3820 (73.2)	< 0.001

Abbreviations: BMI: body mass index; CAD: coronary artery disease; DBP: diastolic blood pressure; DM: diabetes mellitus; eGFR: estimated glomerular filtration rate; FPG: fasting plasma glucose; HbA1c: glycated hemoglobin; HDL-C: high-density lipoprotein cholesterol; IR: insulin resistance; LDL-C: low-density lipoprotein cholesterol; Lp(a): lipoprotein(a); LVEF: left ventricular ejection fraction; NGR: normal glucose regulation; OHA: oral hypoglycemic agents; pre-DM: prediabetes; Rx: prescription; SBP: systolic blood pressure; TC: total cholesterol; TG: triglycerides; TyG: triglyceride-glucose

TyG index	Events (%)	Crude model		Adjusted model	
		Crude OR (95%CI)	P value	Adjusted OR (95%CI)	P value
TyG index as a continuous variable (per 1-unit increment)					
	3696 (70.9)	1.45 (1.3~1.62)	<0.001	1.36 (1.2~1.54)	<0.001
TyG index as a categorical variable					
Q1	855 (65.6)	1(Ref)		1(Ref)	
Q2	893 (68.5)	1.14 (0.97~1.35)	0.107	1.12 (0.95~1.33)	0.181
Q3	950 (72.8)	1.41 (1.19~1.66)	<0.001	1.35 (1.12~1.61)	0.001
Q4	998 (76.6)	1.72 (1.45~2.04)	<0.001	1.57 (1.29~1.9)	<0.001
P for trend			<0.001		<0.001

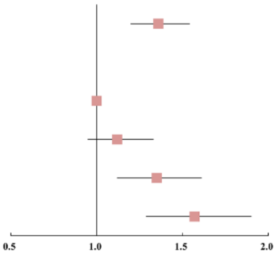

Fig. S1 Association between TyG index and multi-vessel CAD in individuals not using

antilipidemic medications. Adjusted model: adjusted for age, sex, BMI, SBP, current smoking status, hypertension, dyslipidemia, eGFR, LVEF, HDL-C, LDL-C, Lp(a), antidiabetic drugs, antihypertensive drugs, antiplatelet drugs, and antilipidemic drugs. Abbreviations: CI: confidence intervals; OR: odds ratio; TyG: triglyceride-glucose

Subgroup	Events (%)	Crude model		Adjusted model		P value for interaction
		Crude OR (95%CI)	P value	Adjusted OR (95%CI)	P value	
**TyG index as a continuous variable (per 1-unit increment)**						**0.231**
NGR	623 (64.2)	1.44 (1.1~1.89)	0.009	1.47 (1.08~1.99)	0.014	
Pre-DM	1868 (70)	1.35 (1.14~1.61)	0.001	1.31 (1.07~1.59)	0.008	
DM non-insulin Rx	1008 (75.5)	1.31 (1.06~1.63)	0.014	1.41 (1.1~1.79)	0.006	
DM insulin Rx	197 (82.1)	0.95 (0.57~1.58)	0.845	0.81 (0.45~1.46)	0.491	
**TyG index as a categorical variable**						**0.006**
NGR						
Q1	200 (63.5)	1(Ref)		1(Ref)		
Q2	187 (61.1)	0.9 (0.65~1.25)	0.54	0.94 (0.66~1.33)	0.714	
Q3	128 (60.1)	0.87 (0.61~1.24)	0.43	0.78 (0.53~1.14)	0.201	
Q4	108 (79.4)	2.22 (1.38~3.57)	0.001	2.27 (1.35~3.82)	0.002	
P for trend			0.025		0.071	
Pre-DM						
Q1	519 (65.4)	1(Ref)		1(Ref)		
Q2	495 (69.7)	1.22 (0.98~1.51)	0.078	1.18 (0.94~1.48)	0.157	
Q3	505 (73.8)	1.49 (1.19~1.86)	0.001	1.45 (1.14~1.85)	0.003	
Q4	349 (72.3)	1.38 (1.07~1.76)	0.012	1.33 (1.01~1.75)	0.043	
P for trend			0.001		0.008	
DM non-insulin Rx						
Q1	114 (67.5)	1(Ref)		1(Ref)		
Q2	182 (71.7)	1.22 (0.8~1.86)	0.357	1.34 (0.87~2.07)	0.188	
Q3	277 (76.9)	1.61 (1.07~2.41)	0.021	1.89 (1.23~2.9)	0.004	
Q4	435 (78.8)	1.79 (1.23~2.63)	0.003	2.16 (1.41~3.29)	<0.001	
P for trend			0.001		<0.001	
DM insulin Rx						
Q1	22 (81.5)	1(Ref)		1(Ref)		
Q2	29 (87.9)	1.65 (0.4~6.86)	0.493	1.28 (0.26~6.23)	0.757	
Q3	40 (83.3)	1.14 (0.33~3.9)	0.839	0.94 (0.22~3.93)	0.932	
Q4	106 (80.3)	0.93 (0.32~2.68)	0.888	0.7 (0.19~2.56)	0.588	
P for trend			0.529		0.347	

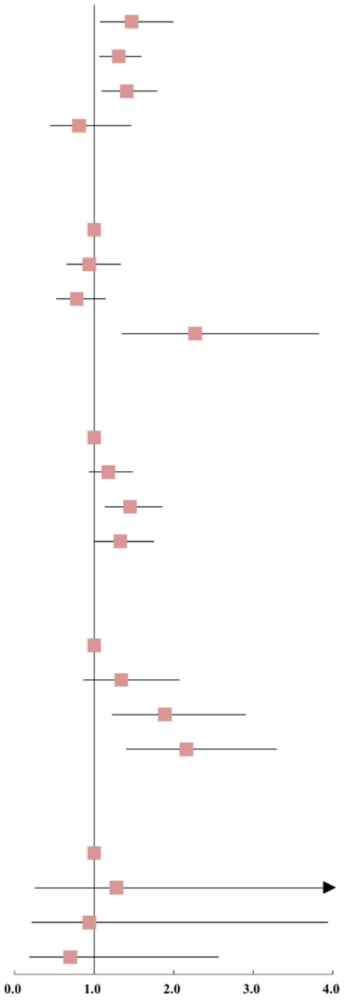

Fig. S2 Glucose metabolism subgroup and interaction analysis of multi-vessel CAD in individuals not using antilipidemic medications. Adjusted model: adjusted for age, sex, BMI, SBP, current smoking status, hypertension, dyslipidemia, eGFR, LVEF, HDL-C, LDL-C, Lp(a), antidiabetic drugs, antihypertensive drugs, antiplatelet drugs, and antilipidemic drugs. Abbreviations: CAD: coronary artery disease; CI: confidence intervals; DM: diabetes

mellitus; NGR: normal glucose regulation; OR: odds ratio; pre-DM: prediabetes; Rx: prescription; TyG: triglyceride-glucose
